# Dietary Supplementation with Algae Powders and Carotenoids Enhances Growth Performance and Tissue-Specific Carotenoid Accumulation in Penaeus Vannamei

**DOI:** 10.3390/ani15111550

**Published:** 2025-05-25

**Authors:** Pujiang Liu, Chengwei Huang, Qian Shen, Qijun Luo, Rui Yang, Haimin Chen, Wei Wu, Juanjuan Chen

**Affiliations:** 1 Collaborative Innovation Center for Zhejiang Marine High-Efficiency and Healthy Aquaculture, Ningbo University, Ningbo 315211, China; pujiangliu0@gmail.com (P.L.); shenqian139331@163.com (Q.S.); luoqijun@nbu.edu.cn (Q.L.); yangrui@nbu.edu.cn (R.Y.); chenhaimin@nbu.edu.cn (H.C.); wuwei@nbu.edu.cn (W.W.); 2 Ningbo Academy of Oceanology and Fisheries, Ningbo 315012, China; eltonbobos@163.com

**Keywords:** algae, feeds, deposition, pigmentation

## Abstract

The pigmentation and coloration of *P. vannamei* are primarily determined by the type and concentration of dietary carotenoids, with carotenoid-rich macroalgae serving as effective dietary supplements to enhance pigment accumulation and improve commercial quality. Five experimental diets were formulated with 3% brown algae (*Saccharina japonica*, SJ group; *Sargassum fusiforme*, SF group), red algae (*Neoporphyra haitanensis*, NH group), or 0.1% purified carotenoids (zeaxanthin, ZT group; fucoxanthin, FX group). The results showed that both macroalgae and carotenoid supplementation significantly enhanced weight gain rate (WGR) and specific growth rate (SGR) compared to the control group, with the zeaxanthin and fucoxanthin groups exhibiting the greatest improvements (1.6-fold and 1.3-fold, respectively). The *N. haitanensis*-supplemented diet, which had the highest carotenoid content, resulted in the most pronounced carotenoid accumulation (2.58-fold increase). Carotenoids were mainly deposited in the exoskeleton, followed by the hepatopancreas, with minimal accumulation in muscle tissue. α-Carotene and β-carotene contributed most to exoskeleton deposition, while lutein and zeaxanthin had weaker effects, and fucoxanthin showed no significant influence. Tissue-specific distribution patterns were observed: α-carotene and β-carotene were localized in the exoskeleton; fucoxanthin and zeaxanthin were found only in the exoskeleton and hepatopancreas, and astaxanthin was present in all three tissues. Furthermore, astaxanthin diesters (C20:5 and C22:6) were primarily detected in the exoskeleton and hepatopancreas, while monoesters (C16:0 and C18:0) were specific to muscle. These findings suggest that targeted supplementation of algal-derived carotenoids can enhance both growth and pigmentation in *P. vannamei*, providing a theoretical basis for the development of functional feeds to improve shrimp commercial quality.

## Simple Summary

Due to its inability to synthesize carotenoids de novo, the Pacific white shrimp (*P. vannamei*) completely depends on dietary sources, making its commercial quality highly feed-dependent. To elucidate the absorption and tissue-specific distribution of algal-derived carotenoids in *P. vannamei*, we conducted analyses of carotenoids profiles in both feeds and different tissues, followed by correlation assessments between dietary intake and tissue deposition patterns. The results demonstrated that dietary carotenoids positively correlated with shrimp carotenoid content, with the exoskeleton as the major deposition site. α/β-carotene dominated exoskeleton pigmentation, while astaxanthin esters showed tissue-specific distribution: diesters in exoskeleton/hepatopancreas and monoesters specifically in muscle. These findings provide critical theoretical insights for optimizing feed formulations to enhance both growth and pigmentation in shrimp.

## 1. Introduction

Carotenoids play vital roles in aquatic animals, including antioxidant, immunoregulatory, and pigmentation functions [1]. Notably, aquatic animals lack the ability to synthesize carotenoids de novo and must obtain them from their diet, subsequently converting dietary carotenes and xanthophylls into tissue-specific carotenoids [2]. Studies have shown that most crustaceans possess the metabolic capacity to convert dietary β-carotene and zeaxanthin into astaxanthin, a major ketocarotenoid, through oxidative reactions at the 4, 4′, 3, and 3′-positions of the β-ionone rings [3]. Dietary supplementation with specific level of carotenoids, such as 100–400 mg/kg β-carotene, has been shown to significantly increase the accumulation of β-carotene, β-cryptoxanthin, echinenone, and astaxanthin in various tissues of *P. vannamei*, including the muscle, head, and exoskeleton [4]. Among these carotenoids, astaxanthin serves as the primary pigment responsible for the coloration observed in crustaceans. In its natural state within the exoskeleton, astaxanthin binds to proteins, forming carotenoproteins, that contribute to the diverse blue, green, purple, and brown hues seen in living crustaceans [5]. Upon cooking, the release of astaxanthin from these protein complexes reveals its characteristic red color. And the concentration of astaxanthin in the exoskeleton exhibits a strong positive correlation with the intensity of the red hue, a critical factor influencing shrimp market value and consumer preference [6]. Consequently, dietary carotenoid supplementation not only enhances the nutritional profile of aquatic species but also improves their market appeal through vibrant coloration.

Micro- and macroalgae are the vital source of carotenoids, which can be classified into three main categories based on their chemical structure: carotenes, xanthophylls, and carotenoids esters [7]. Over the years, algae and carotenoids such as lutein, zeaxanthin, and astaxanthin have gained significant attention in aquaculture as dietary supplements [8,9,10]. For example, the green microalga *Haematococcus pluvialis* is widely used in formulated feeds due to its high content of astaxanthin, which ranges from 1.5% to 3.0% of its dry weight [11]. It effectively increased the astaxanthin content in the muscle, cephalothorax, and shell of *P. vannamei* by 60.10%, 53.72%, and 91.58%, respectively [12]. The carotenoid composition of algae varies significantly due to differences in biochemical characteristics and genetic backgrounds [13,14]. The brown algae, which lack the pathway to synthesize α-carotene, primarily contain fucoxanthin, β-carotene, and violaxanthin. Some red algae, lacking the violaxanthin de-epoxidase (*vde*) gene, predominantly have β-carotene, lutein, and zeaxanthin as their main carotenoids. Green algae, similar to higher plants, mainly contain α-carotene, β-carotene, zeaxanthin, violaxanthin, and neoxanthins [15]. These variations in carotenoid composition may lead to differing effects of algae on the performance of aquatic animals. The rate of pigmentation in shrimp is generally influenced by both the concentration and types of carotenoids in the feed, as well as the duration of feeding [16]. For example, at the same supplementation level (100 mg/kg), the accumulation of astaxanthin in *Penaeus monodon* tissues was 1.23-times and 1.43-times higher than that in shrimp fed with canthaxanthin and β-carotene, respectively [17]. Such functional differences highlight the importance of selecting appropriate algae species based on their carotenoid profiles and carotenoid types to achieve targeted outcomes in aquaculture.

*P. vannamei* is widely regarded as one of the most valuable shrimp species due to its delicious taste and high nutritional value. As a species well-suited to intensive and industrialized farming systems, it plays a pivotal role in global aquaculture, accounting for approximately 70% of the total value of crustacean aquaculture worldwide [18]. In 2022, China’s *P. vannamei* production reached 2.10 million tons, representing a 6.13% increase compared to the previous year, with a mariculture-to-freshwater aquaculture production ratio of 1.76 [19]. With the rapid development of shrimp farming, rising feed costs and reduced profit margin have become one of the major challenges for the industry, driving research into efficient feed formulations, particularly the development and application of feed additives that enhance shrimp growth performance and quality [20]. Previous studies have primarily focused on the effects of algae (mainly microalgae) and carotenoids such as astaxanthin and β-carotene on the immune, antioxidant, and growth responses in *P. vannamei* [21]. Despite the increasing application of algae in shrimp aquaculture, there has been limited research on the relationship between various algae species and carotenoid types as feed additives and their effects on carotenoid deposition in *P. vannamei* [4]. In our present study, dietary supplementation with brown algae (*S. japonica* and *S. fusiforme*), red alga (*N. haitanensis*), and carotenoids (zeaxanthin and fucoxanthin) were compared regarding the carotenoid composition of the feeds on the growth performance and carotenoid accumulation, particular astaxanthin and astaxanthin esters of *P. vannamei*. These findings may provide valuable insights for optimizing feed formulations to enhance the quality and efficiency of pacific white shrimp aquaculture.

## 2. Materials and Methods

### 2.1. Experimental Feeds

The control feed for *P. vannamei* was prepared using the formula detailed in Table 1, with all ingredients sourced from Tech-bank Aquatic Feed Co., Ltd. (Ningbo, China). In addition, five experimental feeds were formulated, each incorporating 3% powders of SJ, SF, or NH and 0.1% carotenoids—either ZX or FX—to replace an equivalent proportion of cellulose in the control feed. The algae powders of SJ and SF were obtained from Xiangshan Xuwen Seaweed Co., Ltd. (Ningbo, China), while NH was sourced from Tengye Seaweed Cultivation Co., Ltd. (Ningbo, China). All algae materials were finely ground and passed through a 100-mesh sieve prior to use. Zeaxanthin and fucoxanthin were obtained from Shanxi Xiazhou Biotechnology Co., Ltd. (Xi’an, China) and Nanjing Tongying Biotechnology Co., Ltd. (Nanjing, China) with a purity exceeding 50%, respectively. The detail feed preparation process followed the methodology described in our previously publication [22]. Finally, all feeds were vacuum-sealed in bags and stored at −20 °C until use.

### 2.2. Shrimp Rearing and Sampling

Juvenile *P. vannamei*, sourced from Chia-Tai Ningbo Company (China), were acclimated for 14 days in a continuously aerated culture system at the Ningbo University test base and fed a control feed. Subsequently, an 8-week feeding trial was conducted using experimental feeds in an indoor flow-through seawater system. A total of 540 juveniles, averaging 2.60 ± 0.20 g in weight, were randomly distributed into eighteen 400 L cylindrical fiberglass tanks (63 cm in diameter × 72 cm in height), each filled with 250 L of seawater, at a stocking density of 30 shrimp per tank. Each feed was randomly assigned to three replicate tanks. The farming management was briefly outlined as described in the previous report [22]. The juveniles were fed with each feed three times daily at 7:00, 12:00, and 18:00, with a daily feeding rate set at 6~8% of their body weight. Before the morning feeding, feces and molts were removed by siphoning the tanks. Seawater conditions were monitored daily using a YSI Proplus device (YSI, Yellow Springs, OH, USA). The recorded environmental parameters were as follows: temperature was 28.48 ± 0.41 °C; salinity, 28.98 ± 0.55‰; pH, 8.0 ± 0.5; ammonia-nitrogen, 0.04 ± 0.002 mg/L; and dissolved oxygen, 6.87 ± 0.24 mg/L. During the cultivation period, 50% of the seawater was exchanged every two days. At the end of the 8-week trial, the shrimp were fasted for 24 h before sampling and then anesthetized with 10 mg/L eugenol (Sinopharm Chemical Reagent Co., Ltd., Shanghai, China). Three shrimp from each tank were randomly selected and dissected on ice to collect tissues, including the second segment of muscle, exoskeleton, and hepatopancreas. Tissues from the same body part in the same tank were pooled together and stored at −80 °C for further carotenoid and fatty acid analysis. Each feed group was represented by three tanks, thus comprising triplicates for each group.

### 2.3. Growth Performance and Colorimetric Analysis

The surface color of 18 shrimp samples from six groups (each in triplicate) was measured using a colorimeter (CS-410, Caipu, Hangzhou, China). The second abdominal segment of each raw shrimp was placed on a standard whiteboard to determine the L* (brightness), a* (redness), and b* (yellowness) values. Thus, the whiteness value was calculated using the following equation [23]:

(1) $$W=100-\sqrt{{\left(100-L*\right)}^{2}+{a*}^{2}+{b*}^{2}}$$

The growth performance was assessed based on the weight of the shrimp. The final body weight (FW), specific growth rate (SGR), weight gain (WG) and survival rate (SR) were calculated using the following equations described by [23].

Body weight gain (WG) = Final weight (g) − Initial weight (g)(2)

Body weight gain rate (WGR, %) = [Final weight (g) − Initial weight (g)]/Initial weight (g) ×100(3)

Specific growth rate (SGR, %/day) = 100 × [ln Final weight (g) − ln Initial weight (g)]/days(4)

Survival rate (SR, %) = (the number of the surviving shrimp/the number of the initial shrimp stock) × 100(5)

### 2.4. Free Carotenoids Analysis in Algae Powders and Feeds

The algae powders and feeds were freeze-dried using liquid nitrogen and then placed in a freeze dryer until they reached a constant weight. The samples were subsequently ground into dry powder under liquid nitrogen. A 100 mg lyophilized sample was weighed into an Eppendorf tube, and 1 mL of cold acetone containing 0.1% BHT was added as the extraction solvent. The mixture was vortexed for 5 min and then subjected to ultrasonic extraction at 500 W for 60 min at 4 °C. After centrifugation (48.3 g, 5 min), the acetone layer was collected. The residue was subjected to the same extraction process three times. The acetone layers were pooled and then concentrated under a stream of nitrogen. The extracts were reconstituted in 1.0 mL of methanol (0.1% formic acid) and filtered through a 0.22 μm PTFE membrane before analysis. The entire process was carried out in the dark and at low temperature (4 °C).

Carotenoid analysis was conducted using an ACQUITY Premier ultra-performance liquid chromatography coupled with a TQ-XS mass spectrometer (UPLC-MS, Waters, MI, USA). A Syncronis C18 column (150 mm × 2.1 mm, 1.7 μm) was employed with the mobile phase consisting of A: acetonitrile (ACN)–methanol (7:3, *v*/*v*) and B: 10 mmol/L ammonium acetate in water. The gradient elution program was as follows: 0–10 min, 70–90% A; 10–15 min, 90% A; 15–18 min, 90–95% A; 18–30 min, 95–100% A; 30–38 min, 100% A; 38–38.1 min, 100–70% A; 38.1–42 min, 70% A. The flow rate was set at 0.3 mL/min, the injection volume was 2 μL, and the column temperature was maintained at 40 °C. The ionization was carried out in positive ion mode using an electrospray ion source (ESI^+^). The capillary voltage was set to 2.0 kV, and the cone voltage was set to 30 V. The desolvation gas (N_2_) temperature was 450 °C, with a flow rate of 1000 L/h. The pressure of the nebulizer gas was set at 7 bar. Argon (Ar) was used as the collision gas, with a collision energy of 6 V. The scanning was performed in multiple reaction monitoring (MRM) mode, as described in a previous report [22]. Carotenoid standards, including α-carotene, β-carotene, ε-carotene, α-cryptoxanthin, β-cryptoxanthin, zeaxanthin, lutein, antheraxanthin, and fucoxanthin (purity > 95%) were purchased from Sigma-Aldrich (St. Louis, MO, USA). Qualitative analysis was performed based on the precursor ions (m/z) and retention times of free carotenoids in the samples, comparing them with those of the standards. The free carotenoid contents in the feeds and algae powders were calculated based on the standard curves, which were constructed by correlating concentration with peak areas using the standard compounds.

### 2.5. Carotenoids Analysis in Shrimps

A five hundred mg freeze-dried sample was ground into a fine powder in liquid nitrogen and then mixed with 10 mL acetone containing 0.1% BHT. The mixture was vortexed for 5 min and subjected to ultrasonic extraction at a power of 500 W for 20 min in an ice bath. The extract was centrifuged at 4830 g for 10 min using a high-speed centrifuge (Centrifuge 5430R, Eppendorf, Germany). The supernatant was collected, concentrated to dryness, and reconstituted with 1 mL acetone containing 0.1% BHT. The reconstituted solution was filtered through a 0.22 μm PTFE membrane and stored for analysis. The entire experimental process was carried out in the dark and at a low temperature (4 °C), with the samples wrapped in aluminum foil to avoid degradation of carotenoids.

Carotenoid analysis was conducted using an ultra-high-performance liquid chromatography coupled with an orbitrap high-resolution mass spectrometer (UHPLC-Q-Orbitrap-HRMS, Thermo Fisher Scientific, Waltham, MA, USA). The separation was performed on a Syncronis C18 column (150 mm × 2.1 mm, 1.7 μm) with the mobile phase consisting of 10 mM ammonium formate in ACN–water (90: 10, *v*/*v*) (A) and ACN–isopropanol (70:30, *v*/*v*) (B). The gradient elution program spanned as follows: 0–5 min, 5% B; 5–15 min, 5–50% B; 15–20 min, 50–100% B; 20–30 min, 100% B; and equilibration at 5% B for 5 min before the next injection. The flow rate was set at 0.3 mL/min, the column temperature was maintained at 35 °C, and the injection volume was 5.0 μL. For ionization, a heated electrospray ionization (H-ESI) source of MS was utilized. Gas flow rates were set at 35 (sheath gas), 10 (auxiliary gas), and 0 (sweep gas) arbitrary units. The spray voltage was maintained at 3.80 kV, while the source and capillary temperatures were 350 °C and 300 °C, respectively. The full MS scan in positive ionization mode was utilized for quantification, with a peak width of 70,000 FWHM at m/z 200, and a scanning range from m/z 100 to m/z 1200. The automatic gain control targeted 3 × 10^6^ ions with a maximum injection time of 200 ms. In addition, data-dependent acquisition (ddMS^2^) scans were employed to generate product ion spectra for qualification, with a resolution of 35,000 FWHM at m/z 200 in MS^2^ mode. Standards, including α-carotene, β-carotene, zeaxanthin, fucoxanthin, astaxanthin, astaxanthin monooleate (18:2), and astaxanthin dipalmitate (16:0/16:0), with purity > 95%, were mixed at a concentration of 10 μg/mL. Sequentially, a series of dilutions were prepared at concentrations of 0.01, 0.02, 0.05, 0.1, 0.2, and 0.5 μg/mL to conduct calibration curves for quantitative or semi-quantitative analysis.

### 2.6. Correlation and Statistical Analysis

Using GraphPad Prism 8, a linear regression analysis was performed to examine the relationship between the carotenoid content in feed and shrimp tissues. First, the carotenoid content in the feed was set as the independent variable (X), and the carotenoid content in shrimp tissues was set as the dependent variable (Y). Subsequently, the linear regression analysis module was selected, with the model configured as simple linear regression, while enabling the Pearson correlation coefficient, 95% confidence interval, and two-tailed significance testing. Upon completion of the analysis, the software output the regression equation (Y = aX + b), the coefficient of determination (R^2^ value), the significance level (*p* value), and the confidence intervals for the slope and intercept. To visually represent the relationship, an XY scatter plot was created, with a regression line added to the graph, along with annotations of the regression equation and R^2^ value.

## 3. Results

### 3.1. Growth Performance and Colorimetric Analysis

As illustrated in Table 2, the three algae powder supplementations positively affected the growth performance of shrimp (*P. vannamei*). Specifically, the final weight WG and WGR of shrimp in the algal-supplemented groups (SJ group, SF group, and NH group) were approximately 1.2 to 1.3 times, 1.2 to 1.3 times, and 1.4 to 1.3 times higher, respectively, compared to the control group. Additionally, the SGR in the algal-supplemented groups was about 1.2 times higher than that in the control group. The supplementation of zeaxanthin and fucoxanthin resulted in more pronounced improvements in growth, with WGR and SGR values approximately 1.65 and 1.3 times higher, respectively, compared to the control group. No significant differences were observed between the carotenoid-supplemented groups in terms of these growth parameters. Moreover, no significant differences in survival rates were observed among the control group, the algal-supplemented groups, and the carotenoid-supplemented groups, except for a statistically significant lower survival rate in the NH group compared to the control group. To investigate the color variation, the surface color parameters of the shrimp fed with different feeds were measured. The supplementation of algae powders and carotenoids all decreased the L*, a* and b* values, except for zeaxanthin, which enhanced the a* value compared to the control group. Additionally, the feeds with added algae powders and carotenoids all led to increases in the W values, indicating a greater overall color change.

### 3.2. Carotenoids Profile in Algae and Feeds

**Figure 1:**
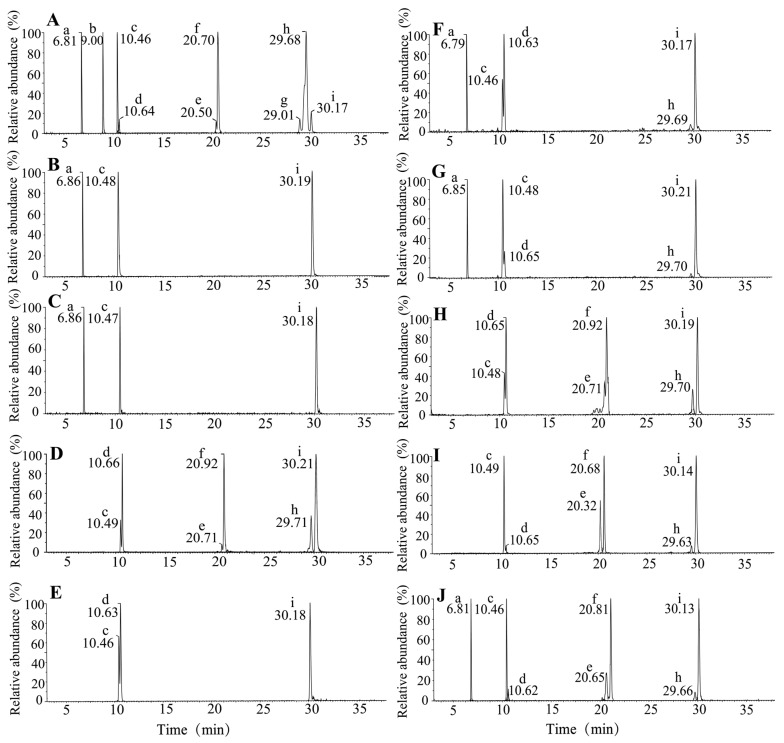
The extracted ion chromatograms (EICs) of carotenoids in algae powders and feeds. The EIC of the carotenoid standards (**A**); the EIC of carotenoids in SJ, SF, and NH powders, respectively (**B**–**D**); the EIC of carotenoids in the control feed (**E**), SJ-supplemented feed (**F**), SF-supplemented feed (**G**), NH-supplemented feed (**H**), ZX- supplemented feed (**I**) and FX-supplemented feed (**J**). Peak a: fucoxanthin; Peak b: antheraxanthin; Peak c: zeaxanthin; Peak d: lutein; Peak e: α-cryptoxanthin; Peak f: β-cryptoxanthin; Peak g: ε-carotene; Peak h: α-carotene; Peak i: β-carotene.

Based on the retention times, precursor ions, product ions, and isotopic distribution compared with available standards, a total of seven types of carotenoids were identified in the powders of SJ, SF, and NH (Figure 1). NH, as a red alga, contained carotenoids related to both the α-carotene pathway—such as α-carotene, α-cryptoxanthin, and lutein—and the β-carotene pathway, including β-carotene, β-cryptoxanthin, and zeaxanthin. In contrast, the brown algae SJ and SF contained only β-carotene pathway-related carotenoids—β-carotene, zeaxanthin, and fucoxanthin. Notably, the total carotenoid content of NH group was 281.66 mg/kg, significantly higher than those of SJ group (18.55 mg/kg) and SF group (13.20 mg/kg) by approximately 15.2 and 21.33 times, respectively (Table 3). Zeaxanthin, lutein, and β-carotene are the primary carotenoids in NH, accounting for 27.1%, 29.2%, and 25.8% of the total carotenoids, respectively. Specifically, fucoxanthin was exclusively detected in both brown algae, which were 19.3% and 18.2% of the total content of carotenoids, with contents of 3.58 mg/kg and 2.40 mg/kg, respectively. The carotenoid levels in most diet groups supplemented with algal powder or fucoxanthin were comparable to those in the control group, except for the NH and ZX groups, which exhibited significantly higher levels. The carotenoid concentrations in the NH and ZX groups were 2.2 and 1.7 times higher than those in the control group, respectively. Specifically, fucoxanthin was only found in the FX, SJ, and SF groups. The highest zeaxanthin content was observed in the ZX group, which was 10.7 times higher than that of the control group. The zeaxanthin content in the FX, NH, and SF groups were 1.5, 3.2, and 2.3 times higher than that in the control group, respectively.

### 3.3. Carotenoids Identification in Shrimp

**Figure 2:**
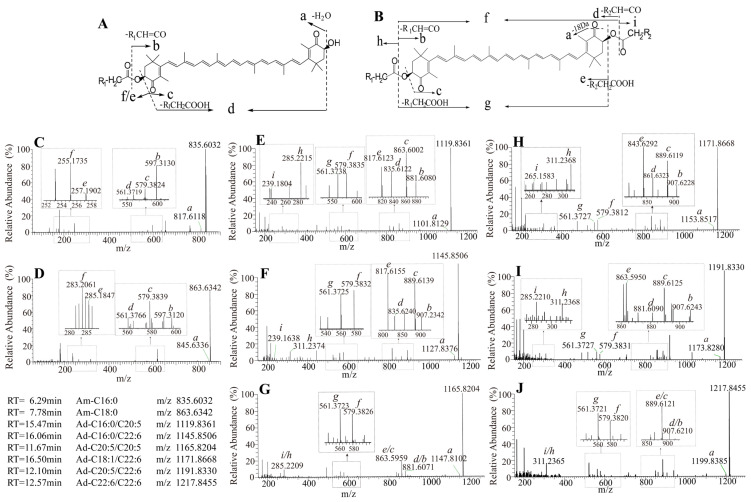
Fragmentation and MS/MS spectra of astaxanthin esters in *P. vannamei*. Fragmentation of the astaxanthin monoester (**A**); fragmentation of the astaxanthin diesters (**B**); the MS/MS spectra of astaxanthin esters: Am-C16:0 (**C**); Am-C18:0 (**D**); Ad-C16:0/C20:5 (**E**); Ad-C16:0/C22:6 (**F**); Ad-C20:5/C20:5 (**G**); Ad-C18:1/C22:6 (**H**); Ad-C20:5/C22:6 (**I**); Ad-C22:6/C22:6 (**J**).

After 8-week feeding traits, free carotenoids including α-carotene, β-carotene, astaxanthin, and eight astaxanthin esters were detected in the shrimp by comparing their retention times, precursor ions, or fragmentation pathways with those of standards. Two types of astaxanthin esters, monoesters and diesters, were identified based on their fragmentation pathways, with astaxanthin monoester (m/z 835.6032) and astaxanthin diester (m/z 1119.8361) subsequently selected as representative examples for structural analysis. In the MS/MS experiment, the astaxanthin monoester exhibited a retention time of 6.29 min with a precursor ion [M+H]^+^ at m/z 835.6032 (Figure 2). The product ions at m/z 817.6118 and 561.3719 were obtained by successive elimination of H_2_O (18 Da) and R_1_CH_2_COOH (256 Da) from the precursor ion. Furthermore, neutral losses of R_1_CH_2_COOH (256 Da) and R_1_CH=CO (238 Da) from the precursor ion generated the product ions at m/z 579.3824 and 597.5233, respectively. These product ions confirmed the presence of the astaxanthin skeleton. The product ions at m/z 255.1735 and 257.1902 indicated the acyl chain, identifying it as palmitic acid C16:0. Therefore, it was identified as an astaxanthin monoester (Am-C16:0). The precursor ion [M + H] ^+^ and product ion [M+H-H_2_O] ^+^ of the astaxanthin diester were observed at m/z 1119.8361 and 1101.8129, respectively. Product ions at m/z 863.6002 and 817.6123 were generated by losses of 256 Da (C16:0, R_1_CH_2_COOH) and 302 Da (C20:5, R_2_CH_2_COOH) from the precursor ion at m/z 1119.8361. Product ions at m/z 881.6080 and 835.6122 were obtained by eliminating 238 Da and 284 Da from the precursor ion, which were assigned to the ketene fragments R_1_CH=CO and R_2_CH=CO, respectively. Successive losses of neutral fragments R_1_CH_2_COOH+R_2_CH_2_COOH (558 Da), and R_1_CH_2_COOH+R_2_CHCO, or R_2_CH_2_COOH+R_1_CHCO (540 Da) yielded product ions at m/z 561.3738 and 579.3835, confirming the astaxanthin skeleton. Additionally, acyl ions expected for fatty acids C20:5 and C16:0 appeared at m/z 285.2215 and 239.1804. Hence, it was putatively identified as an astaxanthin diester (Ad–C16:0/20:5) by the fragmentation pathways and characteristic product ions. Based on the structural analysis described above, a total of eight astaxanthin esters were identified, with their MS/MS spectra and structures shown in Figure 2.

### 3.4. Carotenoid Distribution Across the Shrimp Tissues Influenced by Algal-Supplemented Feed

Based on the calibration curves of α-carotene, β-carotene, free astaxanthin, astaxanthin monooleate (18:2), and astaxanthin dipalmitate (16:0/16:0), three free carotenoids and eight astaxanthin esters were quantified or semi-quantified in the muscle, exoskeleton, and hepatopancreas of *P. vannamei* (Table 4). In the control group, the total contents of carotenoids including both free carotenoids (α-carotene, β-carotene, and astaxanthin) and astaxanthin esters in the exoskeleton, muscle, and hepatopancreas were 33.09 mg/kg, 33.09 mg/kg, and 5.89 mg/kg. Specifically, free carotenoids such as α-carotene and β-carotene were detected only in the exoskeleton. Although free astaxanthin was detected in all three tissues, its content in the exoskeleton was significantly higher than in the hepatopancreas and muscle, with values 12.8 and 44.8 times greater, respectively. However, the proportions of free carotenoids in the shrimp exoskeleton, muscle, and hepatopancreas were low, accounting for only 7.3%, 0.1%, and 0.4% of the total content of carotenoids, respectively, with the majority existing in the form of astaxanthin esters. Astaxanthin diesters were predominantly detected in the exoskeleton (Ad-C20:5/16:0, Ad-C22:6/16:0, Ad-C20:5/20:5, Ad-C22:6/18:1, Ad-C22:6/20:5, and Ad-C22:6/22:6) and hepatopancreas (Ad-C22:6/16:0, Ad-C22:6/18:1, Ad-C22:6/20:5, and Ad-C22:6/22:6), mainly containing C20:5 and C22:6 fatty acyl chains, while astaxanthin monoesters (Am-C16:0 and Am-C18:0) were exclusively found in the muscle, with saturated fatty acid acyl chains. Additionally, although the total carotenoid content was the same in the exoskeleton and muscle, the ratio of astaxanthin esters to free astaxanthin was dramatically different, with values of 7.8 and 412.6, respectively, while the ratio in the hepatopancreas was intermediate, accounting for 20.0.

The abundance of carotenoids in the exoskeleton, muscle, and hepatopancreas was significantly influenced by the supplementation of algae powders in the feed. Incorporating the 3% NH group into the feed mostly enhanced the accumulation of both free carotenoids and astaxanthin esters, increasing their combined levels in the three tissues by approximately 2.6 times compared to the control group. Supplementation of the 3% SF group resulted in a 1.5-fold increase in total carotenoid content. However, no significant difference in carotenoid content was observed between the SJ group and the control group. The accumulation of carotenoids varied across different tissues. Carotenoid accumulation was significantly enhanced in the exoskeleton and hepatopancreas, increasing by 3.7 and 4.8 times in the NH group and 1.6 and 1.8 times in the SF group, respectively, compared to the control group. No significant changes were observed in the muscle of the NH and SJ groups, while the SF group showed a 1.3-fold increase in carotenoid content. Particularly, in the exoskeleton, the contents of α-carotene, β-carotene, and free astaxanthin increased by approximately 9.6, 12.3, and 2.3 times, respectively, with six types of astaxanthin diesters also showing an increase ranging from 1.8 to 4.2 times under the NH-supplemented feed. In the hepatopancreas, the levels of free astaxanthin and four astaxanthin esters (Ad-C22:6/16:0, Ad-C22:6/18:1, Ad-C22:6/20:5, and Ad-C22:6/22:6) significantly increased by approximately 3.5, 25.4, 4.1, 2.2, and 9.0 times, respectively, compared to those in the control group. In the muscle, only free astaxanthin showed an increase, with a rise of 7.3 times in the NH-supplemented group, although the content remained low.

### 3.5. Carotenoid Distribution Across the Shrimp Tissues Influenced by Carotenoid-Supplemented Feed

Fucoxanthin, a characteristic carotenoid found in brown algae, and zeaxanthin, a predominant carotenoid in red algae, were utilized as feed additives to investigate their impact on the carotenoid composition in shrimp (Table 4). Unlike the groups fed with algae powders, direct supplementation of zeaxanthin or fucoxanthin resulted in their specific deposition in the exoskeleton and hepatopancreas, respectively. Furthermore, in the absence of additional α-carotene and β-carotene supplementation, the levels of these two carotenes in the exoskeleton showed no significant difference compared to the control group. Although the levels of certain astaxanthin esters, such as Ad-C20:5/20:5 and Ad-C22:6/22:6, increased in the exoskeleton, the overall effects of fucoxanthin-supplemented feed showed no significant difference compared to the control group and the SJ group. The zeaxanthin-supplemented feed significantly increased the total carotenoid content in shrimp, reaching 2.2 times that of the control group, but it was 85.5% of the level observed in the NH group. Moreover, the carotenoid content in the exoskeleton, hepatopancreas, and muscle was significantly increased, showing 2.7-fold, 3.1-fold, and 1.6-fold enhancements, respectively, compared to the control group. In the ZX group, the composition of free astaxanthin and astaxanthin esters varied across different tissues. The free astaxanthin content in the exoskeleton was significantly lower than that in the NH group (60.5%) but comparable to that in the SF group. Conversely, the free astaxanthin levels in both the muscle and hepatopancreas were significantly higher, reaching 1.1 to 1.3 times those of the NH group. Additionally, most astaxanthin diesters in the exoskeleton and hepatopancreas showed a significant decrease, while astaxanthin monoesters in the muscle exhibited an increase compared to the NH group.

### 3.6. Correlation Analysis Between the Carotenoid Composition of Feeds and Shrimp

**Figure 3:**
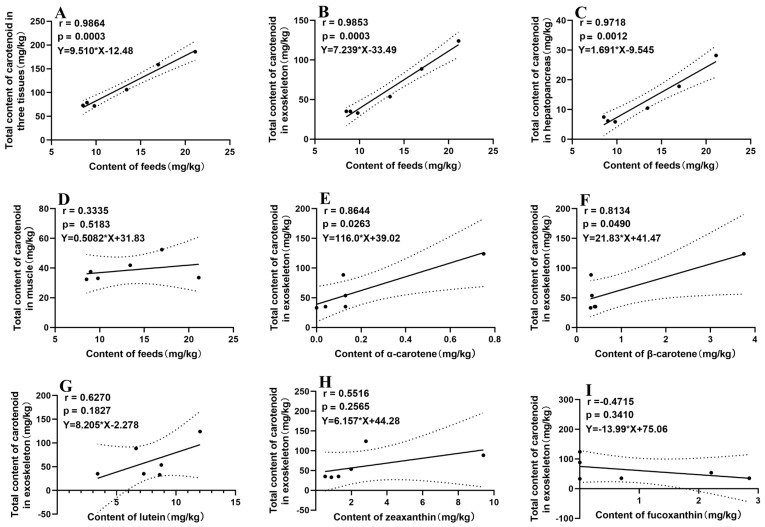
Pearson correlation analysis of the carotenoid contents between feeds and *P. vannamei*. The correlation between the total content of carotenoids in the three tissues and feeds (**A**); the correlation between the total content of carotenoids in the exoskeleton (**B**), hepatopancreas (**C**), muscle (**D**), and the content of the feeds, respectively. The correlation between the total content of carotenoids in the exoskeleton and the content of α-carotenoid (**E**), β-carotene (**F**), lutein (**G**), zeaxanthin (**H**), and fucoxanthin (**I**) in the feeds, respectively.

As shown in Figure 3, the Pearson correlation analysis showed that the linear relationship between the dietary carotenoid content and carotenoids accumulation in the three tissues (exoskeleton, hepatopancreas, and muscle) of *P. vannamei* was highly correlated and significant (*p* < 0.05). The overall relationship was described by the equation Y = 9.51X − 12.48, with a correlation coefficient of r = 0.9864. The slope of the regression equation reflected the rate of carotenoid deposition in the total tissues in response to dietary carotenoid content, revealing that for every 1-unit increase in dietary carotenoid content, the total carotenoid level in the shrimp tissues increased by 9.51 units. In addition, the influence of dietary carotenoid levels on carotenoid accumulation in different tissues of *P. vannamei* revealed tissue-specific responses. The exoskeleton and hepatopancreas exhibited significant positive correlations (*p* < 0.05) with the linear regression equation of Y = 7.24X − 33.49 (r = 0.99) and =1.69X − 9.55 (r = 0.97), respectively. In contrast, the muscle tissue showed a weaker (Y = 0.51X + 31.83, r = 0.33) and non-significant response (*p* > 0.05). The slopes of the regression equations suggested that the exoskeleton was the primary site of the carotenoid deposition, followed by the hepatopancreas, whereas the muscle tissue showed minimal deposition. Furthermore, the effects of typical carotenoids, including α-carotene, β-carotene, lutein, zeaxanthin, and fucoxanthin, on carotenoid deposition in the exoskeleton of shrimp were compared. The results indicated that the accumulation of total carotenoids in the shrimp exoskeleton was strongly correlated with the presence of α-carotene and β-carotene in the feed, as evidenced by a linear correlation coefficient (r) greater than 0.8. This suggested that these two carotenoids played a significant role in enhancing the total carotenoid content in the exoskeleton. On the other hand, lutein and zeaxanthin showed a moderate linear correlation with the total carotenoid deposition, with correlation coefficients ranging between 0.5 and 0.8. This implies that while these carotenoids did contribute to the total carotenoid content in the exoskeleton, their influence was not as strong as that of α-carotene and β-carotene.

## 4. Discussion

Carotenoids possess a variety of biological functions, which were beneficial for growth and pigmentation in aquatic animals [4]. Studies have shown the positive effects of dietary carotenoids on the growth performance in shrimp when the supplemented carotenoids content exceeded 100 mg/kg, with astaxanthin exhibiting significant effects. In contrast, lower concentrations did not produce noticeable effects [24,25,26]. However, the dosage suggested by many publications only specified the amount of carotenoid added into the feed. During the feed production process, carotenoids with long-conjugated system of double bonds were prone to degradation into volatile or apo-carotenoids due to the factors such as high temperature and light [27,28]. This degradation resulted in a reduction in the actual carotenoid content in the feed. Lin et al. reported that the actual concentration of astaxanthin in the feed for *P. vannamei* was reduced by nearly half compared to the theoretical amount [6]. Therefore, the actual carotenoid content in the feed might differ significantly from the theoretically added amount. Here, we explored the effects of a low amount of carotenoid on shrimp farming.

Favorable growth performance in crustaceans is widely regarded as the primary aim of aquaculture. Seaweeds were often utilized as dietary supplements due to their abundant bioactive compounds and nutrients, which were beneficial for enhancing growth performance in crustaceans by promoting well-developed intestinal structure and improving digestive and absorptive capacity [22]. The present study supported the positive effect of algae powder supplementation on the growth performance, although no significant impact on survival was observed in *P. vannamei*. The carotenoid content in SF-, SJ-, and NH-supplemented feeds gradually increased, yet despite this, no significant difference in WGR and SGR were observed among these groups. This suggested that, in addition to carotenoids, other nutritional components in the algae might also play roles in regulating growth performance, making it difficult to clearly assess the specific effect of carotenoids on growth performance. It was well established that carotenoids, including well-known antioxidants such as astaxanthin and β-carotene when supplemented in the feed, could enhance the growth and survival of *P. vannamei* [29]. Comparing to the control and SJ-supplemented feeds, the content of carotenoids in NH- and SF-supplemented feeds were higher. The NH-supplemented feed contained abundant β-carotene, lutein, and zeaxanthin, while the SF-supplemented feed was rich in zeaxanthin and fucoxanthin. Different carotenoids exhibited varying effects on growth performance. For example, the growth performance and survival of *P. monodon* fed with 0.25% β-carotene were comparable to those fed with 0.1% astaxanthin [25]. Given that previous studies have investigated the effects of β-carotene and lutein on shrimp growth, this study aimed to evaluate the effects of zeaxanthin and fucoxanthin [30]. Clearly, the effect of supplementing these two carotenoids in the feed was more significant than that of the algae powders. This might be because that the algae powders must be digested first before free-form carotenoids released, whereas zeaxanthin and fucoxanthin were directly utilized by shrimp and strengthened the growth performance [31].

Body color is also a determinant of the quality and market value of shrimp, primarily due to the presence of various carotenoids that contribute to the pigmentation. However, crustaceans lack the enzymatic machinery required for de novo synthesis of carotenoids [32]. Instead, they must obtain carotenoids through diets and inter-convert into different types of carotenoids within their body [33,34]. Beyond their role in pigmentation, carotenoids also exert a range of physiological benefits in shrimp. They have been shown to shorten molting cycle intervals, enhance digestive efficiency, and improve stress tolerance, thereby facilitating nutrient accumulation and rapid growth, which ultimately contribute to improved overall growth performance [16,35,36]. Following ingestion, dietary carotenoids such as α-carotene, β-carotene, lutein, zeaxanthin, and astaxanthin are either stored in their native form or converted into astaxanthin through processes like ketolation and hydroxylation, originating from β-carotene or zeaxanthin as the precursors [37]. Consistent with these findings, the accumulation of carotenoids—particularly astaxanthin—and associated color parameters demonstrated significant variations in response to dietary supplementation with algae powders and carotenoid-enriched feeds. Studies on *P. monodon* have further demonstrated that when astaxanthin reached a certain threshold concentration in shrimp fed with *Spirulina*-supplemented diets, the metabolic conversion of its precursors was inhibited, leading to their deposition in tissues [38]. In the present study, β-carotene was predominantly detected in the exoskeleton of the NH group, while zeaxanthin was exclusively observed in the exoskeleton and hepatopancreas of the ZT group, both of which exhibited higher levels of free astaxanthin. For raw shrimp, the L*, a*, and b* values were lower in the algal-/carotenoid-supplemented groups which indicated that the body color became darker, greener, and bluer, appearing as a dark grey color [39]. This finding was consistent with previous reports that carotenoid supplementation resulted in darker raw shrimp and a more reddish color after cooking [1]. The shrimp raw color is related to the formation of the crustacyanin, a protein–astaxanthin complex, with higher content of astaxanthin resulting in a darker color. After cooking, this protein–astaxanthin complex would be dissociated and free astaxanthin is released to provide the vibrant color. In this study, the darker color of *P. vannamei* was associated with the higher content of carotenoids, particularly astaxanthin, after feeding with dietary algae powders and zeaxanthin supplementation [40].

Previous studies have reported that the increased levels of dietary carotenoid led to the accumulation of carotenoids in crustaceans [35]. In the current experiment, Pearson correlation analysis confirmed a positive correlation between carotenoid content in the feed and that in shrimp. Specifically, each unit increase in dietary carotenoids resulted in an approximate 9.51-unit increase in the carotenoid content of shrimp. Similar patterns of carotenoid deposition were observed in the exoskeleton and hepatopancreas, whereas no significant correlation was detected in the muscle. This phenomenon could be explained by the fact that the hepatopancreas serves as the primary site for the digestion and metabolic conversion of carotenoids into astaxanthin in crustaceans. Subsequently, these compounds are deposited readily in tissues such as the exoskeleton and ovaries [41]. In contrast, the deposition efficiency of carotenoids in the muscle is lower compared to other tissues, likely due to a reduced absorption rate in the gastrointestinal tract and some carotenoids are metabolized into colorless compounds, such as vitamin A derivatives [24,42]. Moreover, different polarity and properties of carotenoids showed different effects on the carotenoid deposition in the exoskeleton. In the present study, nonpolar hydrocarbon carotenes, such as α-carotene and β-carotene, exhibited strong linear correlations, which could be attributed to the highest carotenoid content in shrimp. In contrast, lutein, zeaxanthin, and fucoxanthin displayed weak correlations. This might be because β-carotene acts as a critical precursor for astaxanthin formation in shrimp [4]. The elevated levels of α-carotene and β-carotene in the NH-supplemented feed correlate with increased astaxanthin content, encompassing both free astaxanthin and its esterified forms in the exoskeleton.

The analytical results demonstrated a pronounced tissue-specific distribution of free astaxanthin, with the highest concentration localized in the exoskeleton, while only trace levels were detected in the hepatopancreas and muscle tissues. This distribution pattern might be attributed to the exoskeleton’s role as the primary defensive barrier against environmental stressors, where astaxanthin is strategically utilized for its potent antioxidative properties. Additionally, free astaxanthin in the exoskeleton plays an essential role in pigmentation, enabling background color matching. Moreover, the content of free astaxanthin is dynamically governed by esterification-mediated metabolic regulation [43]. Studies have consistently demonstrated that astaxanthin binds with fatty acids of varying chain lengths to form monoesters or diesters, which serve as stabilized storage forms within tissues [43,44,45]. Notably, astaxanthin monoesters esterified with saturated fatty acids and astaxanthin diesters conjugated with unsaturated fatty acids exhibited superior thermal stability [46]. In *P. vannamei*, the predominant fatty acids that have been identified include C16:0, C18:0, C18:2n6, C20:5n3, and C22:6n3. These explained the observed predominance of astaxanthin diesters primarily composed of C20:5 and C22:6, and astaxanthin monoesters exclusively containing saturated fatty acids (C16:0 and C18:0) in *P. vannamei* [47]. Furthermore, compared to astaxanthin monoesters, astaxanthin diesters contain two longer-chain fatty acids, which exhibit stronger lipophilicity and lower polarity. This structural feature enables the diester form to integrate more readily into membrane structures and need more energy to break up, thereby demonstrating superior thermal stability. In the present study, astaxanthin diesters (primarily C20:5 and C22:6) were predominantly detected in the exoskeleton and hepatopancreas, while astaxanthin monoesters exclusively containing saturated fatty acids (C16:0 and C18:0) were identified in the muscle tissue. These stabilization strategies enable long-term preservation of astaxanthin’s bioactivity, particularly in its di-esterified forms within the exoskeleton for antioxidant defense and coloration demands [48].

## 5. Conclusions

This study represents the first application of UPLC-MS technology to investigate the effects of various algal powders and carotenoids as dietary supplements on growth performance and pigment deposition in *P. vannamei*. The results demonstrated that all five dietary supplements enhanced the growth performance of shrimp. A significant positive correlation was observed between dietary carotenoid content and their deposition in shrimp tissues, with the exoskeleton serving as the primary accumulation site. Notably, *N*. *haitanensis* powder and zeaxanthin exhibited the most pronounced enhancement of carotenoid accumulation, identified as the most effective algal powder and carotenoid additive, respectively. Furthermore, tissue-specific carotenoid accumulation was found to be mediated through free carotenoids and astaxanthin esters. These findings suggest that targeted carotenoid deposition through specific algal species or carotenoid compounds can effectively improve shrimp pigmentation. Future research will focus on elucidating the biosynthetic pathways and metabolic transformation mechanisms of carotenoids in shrimp, aiming to optimize feed formulations and enhance pigmentation outcomes through systematic biological regulation.

## Figures and Tables

**Table 1:** The formula of the feeds for *P. vannamei*.

Ingredients (%)	Control	SJ	SF	NH	ZX	FX
Peruvian fish meal	17.00	17.00	17.00	17.00	17.00	17.00
Domestic fish meal	8.00	8.00	8.00	8.00	8.00	8.00
Shrimp meal	4.00	4.00	4.00	4.00	4.00	4.00
Yeast powder	4.00	4.00	4.00	4.00	4.00	4.00
Soybean meal	18.00	18.00	18.00	18.00	18.00	18.00
Peanut meal	8.00	8.00	8.00	8.00	8.00	8.00
Concentrated protein ^a^	5.00	5.00	5.00	5.00	5.00	5.00
Flour	19.97	19.97	19.97	19.97	19.97	19.97
Cholesterol	0.5.	0.50	0.50	0.50	0.50	0.50
Fish oil	2.00	2.00	2.00	2.00	2.00	2.00
Soybean oil	1.00	1.00	1.00	1.00	1.00	1.00
Phospholipids	2.00	2.00	2.00	2.00	2.00	2.00
Dicalcium phosphate	2.00	2.00	2.00	2.00	2.00	2.00
Choline chloride	0.50	0.50	0.50	0.50	0.50	0.50
Antioxidants	0.03	0.03	0.03	0.03	0.03	0.03
Vitamin premix ^b^	1.00	1.00	1.00	1.00	1.00	1.00
Mineral mixture ^c^	2.00	2.00	2.00	2.00	2.00	2.00
Cellulose	5.00	2.00	2.00	2.00	4.90	4.90
Algae product meal	0.00	3.00	3.00	3.00	0.10	0.10
Total	100.00	100.00	100.00	100.00	100.00	100.00

^a^ Soy Protein Concentrate. ^b^ Vitamin premix (mg/kg dry diet): D-Ca pantothenate, 120; Inositol, 200; Menadione, 60; Nicotinic acid, 100; Pyridoxine hydrochloride, 60; Riboflavin, 50; Thiamin nitrate, 60; All-rac-a-tocopherol, 100; Cyanocobalamin, 0.1; Biotin, 6.0; Folic acid, 10; Retinyl acetate 5000 IU; Cholecalciferol, 2000 IU. ^c^ Mineral mixture (g/kg mineral premix): C_6_H_10_CaO_6_·5H_2_O (99.5%), 77.83; KCl (99.5%), 191.62; MgSO_4_·7H_2_O (99%), 614.48; NaCl (99.5%), 76.69; C_6_H_5_O_7_Fe·5H_2_O, 6.8571; CuSO_4_·5H_2_O, 10.2041; ZnSO_4_·7H_2_O, 9.2754; Co-Met, 5.0; MnSO_4_·H_2_O, 6.2893; KI (0.99%), 1.7034; Na_2_SeSO_3_, 0.5.

**Table 2:** Growth performance and colorimetric parameters of *P. vannamei* with different feed supplements.

Index	Control	SJ	SF	NH	ZT	FX
Initial weight (g)	2.70 ± 0.09 ^a^	2.51 ± 0.09 ^a^	2.49 ± 0.13 ^a^	2.71 ± 0.37 ^a^	2.45 ± 0.06 ^a^	2.48 ± 0.10 ^a^
Final weight (g)	11.22 ± 1.08 ^b^	13.08 ± 1.10 ^ab^	13.03 ± 1.59 ^ab^	14.22 ± 2.24 ^a^	15.41 ± 1.04 ^a^	15.11 ± 0.58 ^a^
WG (g)	8.52 ± 1.04 ^c^	10.57 ± 1.01 ^b^	10.54 ± 1.47 ^b^	11.51 ± 1.88 ^ab^	12.96 ± 0.98 ^a^	12.63 ± 0.48 ^ab^
WGR (%)	315.62 ± 28.86 ^c^	421.12 ± 8.19 ^b^	423.29 ± 14.13 ^b^	424.72 ± 12.93 ^b^	528.98 ± 19.17 ^a^	509.27 ± 10.34 ^a^
SGR (%)	2.37 ± 0.15 ^c^	2.75 ± 0.02 ^b^	2.75 ± 0.04 ^b^	2.76 ± 0.05 ^b^	3.06 ± 0.05 ^a^	3.01 ± 0.03 ^a^
Survival rate (%)	64.29 ± 4.29 ^a^	56.67 ± 4.71 ^ab^	68.33 ± 15.46 ^a^	46.67 ± 5.87 ^b^	61.67 ± 2.36 ^ab^	53.33 ± 12.47 ^ab^
L*	37.63 ± 0.46 ^a^	35.82 ± 0.66 ^b^	35.68 ± 0.24 ^b^	36.01 ± 0.53 ^b^	35.55 ± 0.44 ^b^	36.04 ± 0.41 ^b^
a*	−0.42 ± 0.09 ^b^	−1.14 ± 0.13 ^e^	−1.13 ± 0.05 ^e^	−0.53 ± 0.01 ^c^	−0.29 ± 0.06 ^a^	−0.69 ± 0.02 ^d^
b*	5.21 ± 0.26 ^a^	3.71 ± 0.36 ^c^	3.87 ± 0.03 ^c^	4.50 ± 0.12 ^b^	4.37 ± 0.26 ^b^	4.01 ± 0.15 ^c^
W	62.59 ± 0.44 ^b^	64.29 ± 0.67 ^a^	64.23 ± 0.55 ^a^	64.15 ± 0.52 ^a^	64.60 ± 0.43 ^a^	64.09 ± 0.41 ^a^

WG: weight gain; WGR: body weight gain rate; SGR: specific growth rate. L*: brightness; a*: redness; b*: yellowness. Values in the same row with different low ercase letters (a, b, c, etc.) indicate significant differences (*p* < 0.05), while values sharing at least one common letter (e.g., a and ab) are not significantly different (*p* > 0.05).

**Table 3:** The contents of carotenoid in algae powders and feeds.

Carotenoids	Algae Powder (mg/kg)			Feeds (mg/kg)					
	*S. japonica*	*S. fusiforme*	*N. haitanensis*	Control	SJ	SF	NH	ZX	FX
α-carotene	ND	ND	17.29 ± 0.62 ^a^	ND	0.04 ± 0.002 ^c^	0.13 ± 0.02 ^c^	0.75 ± 0.08 ^b^	0.12 ± 0.01 ^c^	0.13 ± 0.02 ^c^
β-carotene	3.70 ± 0.15 ^b^	5.23 ± 0.25 ^b^	72.62 ± 3.80 ^a^	0.31 ± 0.09 ^c^	0.42 ± 0.04 ^c^	0.34 ± 0.1 ^c^	3.75 ± 0.45 ^b^	0.32 ± 0.04 ^c^	0.40 ± 0.1 ^c^
α-cryptoxanthin	ND	ND	2.20 ± 0.32 ^a^	ND	ND	ND	0.76 ± 0.08 ^b^	0.22 ± 0.02 ^c^	0.24 ± 0.04 ^c^
β-cryptoxanthin	ND	ND	31.11 ± 1.23 ^a^	ND	ND	ND	1.02 ± 0.17 ^b^	0.26 ± 0.06 ^c^	0.23 ± 0.04 ^c^
leutin	ND	ND	82.16 ± 4.12 ^a^	8.62 ± 0.30 ^c^	7.29 ± 0.03 ^c^	8.76 ± 0.94 ^c^	12.02 ± 0.03 ^b^	6.64 ± 0.81 ^c^	3.40 ± 0.69 ^d^
zeaxanthin	11.27 ± 0.44 ^b^	5.62 ± 0.14 ^d^	76.28 ± 0.80 ^a^	0.88 ± 0.08 ^g^	0.53 ± 0.04 ^h^	1.99 ± 0.07 ^f^	2.82 ± 0.02 ^e^	9.41 ± 0.80 ^c^	1.28 ± 0.15 ^g^
antheraxanthin	ND	ND	ND	ND	ND	ND	ND	ND	ND
fucoxanthin	3.58 ± 0.09 ^a^	2.40 ± 0.36 ^bc^	ND	ND	0.70 ± 0.08 ^d^	2.21 ± 0.47 ^c^	ND	ND	2.85 ± 0.18 ^b^
Total	18.55 ± 0.40 ^b^	13.20 ± 0.41 ^c^	281.66 ± 7.53 ^a^	9.81 ± 0.32 ^cd^	8.98 ± 0.03 ^d^	13.43 ± 0.93 ^c^	21.12 ± 0.45 ^b^	16.97 ± 0.14 ^b^	8.53 ± 0.75 ^d^

Values in the same row with different low ercase letters (a, b, c, etc.) indicate significant differences (*p* < 0.05), while values sharing at least one common letter (e.g., a and ab) are not significantly different (*p* > 0.05).

**Table 4:** Carotenoid distribution in different tissues of *P. vannamei* under different dietary supplementations.

Tissue	Carotenoids	Control	SJ	SF	NH	ZT	FX
		(mg/kg)	(mg/kg)	(mg/kg)	(mg/kg)	(mg/kg)	(mg/kg)
Exoskeleton	α-carotene	0.81 ± 0.02 ^c^	1.48 ± 0.12 ^b^	1.74 ± 0.23 ^b^	7.74 ± 1.32 ^a^	0.86 ± 0.06 ^c^	0.81 ± 0.06 ^c^
	β-carotene	0.92 ± 0.07 ^d^	1.68 ± 0.05 ^c^	2.74 ± 0.12 ^b^	11.35 ± 0.35 ^a^	0.94 ± 0.04 ^d^	0.81 ± 0.03 ^d^
	Free astaxanthin	3.58 ± 0.15 ^d^	4.16 ± 0.04 ^c^	4.94 ± 0.07 ^b^	8.12 ± 0.41 ^a^	4.91 ± 0.3 ^b^	3.30 ± 0.34 ^d^
	Zeaxanthin	ND	ND	ND	ND	2.76 ± 0.12 ^a^	ND
	Fucoxanthin	ND	ND	ND	ND	ND	1.35 ± 0.14 ^a^
	Ad-C20:5/16:0	3.05 ± 0.05 ^b^	3.59 ± 0.12 ^b^	5.12 ± 0.22 ^a^	5.37 ± 0.54 ^a^	5.39 ± 0.53 ^a^	3.15 ± 0.05 ^b^
	Ad-C22:6/16:0	6.85 ± 0.35 ^d^	6.67 ± 0.23 ^d^	12.35 ± 1.06 ^c^	24.35 ± 0.86 ^a^	16.19 ± 1.43 ^b^	6.85 ± 0.35 ^d^
	Ad-C20:5/20:5	2.62 ± 0.59 ^d^	2.85 ± 0.09 ^d^	2.21 ± 0.13 ^d^	10.95 ± 0.25 ^b^	12.35 ± 0.5 ^a^	3.95 ± 0.99 ^c^
	Ad-C22:6/18:1	3.72 ± 0.15 ^c^	2.95 ± 0.18 ^c^	5.42 ± 0.43 ^b^	13.85 ± 1.16 ^a^	13.72 ± 0.15 ^a^	3.05 ± 0.59 ^c^
	Ad-C22:6/20:5	6.82 ± 0.07 ^de^	7.43 ± 0.31 ^d^	9.37 ± 0.19 ^c^	23.88 ± 0.59 ^a^	16.82 ± 0.07 ^b^	6.16 ± 1.46 ^e^
	Ad-C22:6/22:6	4.72 ± 1.00 ^e^	4.25 ± 0.16 ^e^	9.85 ± 0.17 ^c^	18.46 ± 0.55 ^a^	14.72 ± 0.23 ^b^	5.72 ± 0.23 ^d^
	Total content	33.09 ± 1.00 ^d^	35.06 ± 0.51 ^d^	53.74 ± 0.67 ^c^	124.07 ± 3.94 ^a^	88.66 ± 1.96 ^b^	35.15 ± 1.67 ^d^
Hepatopancreas	Free astaxanthin	0.28 ± 0.03 ^d^	0.42 ± 0.02 ^c^	0.25 ± 0.04 ^d^	0.98 ± 0.02 ^b^	1.08 ± 0.13 ^a^	0.32 ± 0.04 ^c^
	Zeaxanthin	ND	ND	ND	ND	1.19 ± 0.21 ^a^	ND
	Fucoxanthin	ND	ND	ND	ND	ND	1.20 ± 0.10 ^a^
	Ad-C22:6/16:0	0.14 ± 0.01 ^d^	1.18 ± 0.18 ^c^	1.69 ± 0.18 ^b^	3.55 ± 0.31 ^a^	3.46 ± 0.41 ^a^	0.44 ± 0.11 ^d^
	Ad-C22:6/18:1	2.15 ± 0.38 ^c^	0.51 ± 0.07 ^d^	1.04 ± 0.13 ^d^	8.72 ± 0.83 ^a^	4.46 ± 0.74 ^b^	2.15 ± 0.11^c^
	Ad-C22:6/20:5	2.18 ± 0.12 ^c^	2.14 ± 0.15 ^c^	3.18 ± 0.21 ^b^	4.68 ± 0.73 ^a^	4.4 ± 0.62 ^a^	2.27 ± 0.55 ^c^
	Ad-C22:6/22:6	1.14 ± 0.08 ^c^	1.93 ± 0.25 ^c^	4.32 ± 0.25 ^b^	10.28 ± 1.05 ^a^	3.23 ± 1.44 ^b^	1.10 ± 0.08 ^c^
	Total content	5.89 ± 0.41 ^e^	6.18 ± 0.41 ^d^	10.48 ± 1.08 ^c^	28.21 ± 1.95 ^a^	17.82 ± 0.07 ^b^	7.48 ± 0.6 ^d^
Muscle	Free astaxanthin	0.08 ± 0.01 ^d^	0.21 ± 0.04 ^d^	0.42 ± 0.08 ^c^	0.58 ± 0.01 ^b^	0.78 ± 0.21 ^a^	0.11 ± 0.01 ^d^
	Am-C16:0	14.79 ± 0.29 ^bc^	17.22 ± 2.41 ^b^	19.25 ± 3.02 ^b^	14.79 ± 0.29 ^bc^	24.79 ± 0.29 ^a^	13.79 ± 1.87 ^c^
	Am-C18:0	18.22 ± 2.19 ^b^	20.11 ± 2.15 ^b^	22.25 ± 3.33 ^ab^	18.22 ± 2.19 ^b^	26.89 ± 1.73 ^a^	18.56 ± 2.57 ^b^
	Total content	33.09 ± 2.07 ^c^	37.55 ± 3.34 ^bc^	41.92 ± 4.82 ^b^	33.59 ± 2.07 ^c^	52.46 ± 1.66 ^a^	32.46 ± 2.41 ^c^
Total content in three tissues		72.07 ± 2.07 ^d^	78.79 ± 4.26 ^d^	106.14 ± 6.57 ^c^	185.87 ± 1.96 ^a^	158.94 ± 3.14 ^b^	73.09 ± 3.38 ^d^

Values in the same row with different low ercase letters (a, b, c, etc.) indicate significant differences (*p* < 0.05), while values sharing at least one common letter (e.g., a and ab) are not significantly different (*p* > 0.05).

## Data Availability

The data presented in this study are available in this manuscript.
